# Metastasizing, Luciferase Transduced MAT-Lu Rat Prostate Cancer Models: Follow up of Bolus and Metronomic Therapy with Doxorubicin as Model Drug

**DOI:** 10.3390/cancers3022679

**Published:** 2011-06-17

**Authors:** Peter Jantscheff, Norbert Esser, Andreas Geipel, Peter Woias, Vittorio Ziroli, Frank Goldschmidtboing, Ulrich Massing

**Affiliations:** 1 Tumour Biology Center, Clinical Research, Department Lipids & Liposomes, Breisacher Str.117, D-79106 Freiburg, Germany; E-Mails: ziroli@tumorbio.uni-freiburg.de (V.Z.); massing@tumorbio.uni-freiburg.de (U.M.); 2 ProQinase GmbH, Breisacher Str. 117, D-79106 Freiburg, Germany; E-Mail: n.esser@proqinase.com; 3 Laboratory for Design of Microsystems, Department of Microsystems Engineering (IMTEK), Georges-Köhler-Allee 106, D-79110 Freiburg, Germany; E-Mails: ageipel@imtek.de (A.G.); woias@imtek.de (P.W.); fgoldsch@imtek.de (F.G.)

**Keywords:** prostate cancer, MAT-Lu, orthotopic, bioluminescence, luciferase, rat, dunning

## Abstract

The most fatal outcomes of prostate carcinoma (PCa) result from hormone-refractory variants of the tumor, especially from metastatic spread rather than from primary tumor burden. The goal of the study was to establish and apply rat MAT-Lu prostate cancer tumor models for improved non-invasive live follow up of tumor growth and metastasis by *in vivo* bioluminescence. We established luciferase transduced MAT-Lu rat PCa cells and studied tumor growth and metastatic processes in an ectopic as well as orthotopic setting. An intravenous bolus treatment with doxorubicin was used to demonstrate the basic applicability of *in vivo* imaging to follow up therapeutic intervention in these models. *In vitro* analysis of tissue homogenates confirmed major metastatic spread of subcutaneous tumors into the lung. Our sensitive method, however, for the first time detects metastasis also in lymph node (11/24), spleen (3/24), kidney (4/24), liver (5/24), and bone tissue (femur or spinal cord - 5/20 and 12/20, respectively). Preliminary data of orthotopic implantation (three animals) showed metastatic invasion to investigated organs in all animals but with varying preference (e.g., to lymph nodes). Intravenous bolus treatment of MAT-Lu PCa with doxorubicin reduced subcutaneous tumor growth by about 50% and the number of animals affected by metastatic lesions in lymph nodes (0/4), lung (3/6) or lumbar spine (0/2), as determined by *in vivo* imaging and *in vitro* analysis. Additionally, the possible applicability of the luciferase transduced MAT-Lu model(s) to study basic principles of metronomic therapies via jugular vein catheter, using newly established active microport pumping systems, is presented.

## 1. Introduction

Mortality in prostate carcinoma (PCa) is mostly due to the hormone-refractory metastasizing variant of the tumor. Therefore, since metastatic spread, and not primary tumor burden, seems to be the leading cause of cancer deaths [1], it is important to use well-characterized, clinically relevant metastatic animal models for the investigation of factors that regulate metastatic outgrowth and for the development of new systemic therapies [1].

Chemotherapy is the standard treatment option for palliation of symptoms associated with androgen-independent PCa [2-4]. However, the multidrug-resistant (MDR) nature of advanced PCa minimizes the effectiveness of such therapies, and consequently, most patients die within 12–16 months [5-9]. The search for efficient drugs and treatment schedules is therefore vital to improve therapeutic outcome.

Androgen independent Dunning rat prostate cancer cells, AT-1, AT-3.1, MAT-Lu or MAT LyLu consistently show a clearly augmented resistance to various cytotoxic drugs resulting from increased activity of the rat mdr1b gene, the homologue to the human MDR1 gene [10]. Additionally, they display distinct and strong metastatic capacities [11] and thus behave similar to advanced human prostate cancer cells, and are valuable models for experimental *in vivo* studies in rats.

Bioluminescent transduction of tumor cells recently has been shown to offer the possibility to follow up tumor growth in viable animals in ectopic as well as orthotopic models [12,13] and to sensitively detect metastatic lesions [13-15].

The goal of the present study was to establish luciferase transduced MAT-Lu cells for improved follow up of tumor growth in ectopic subcutaneous as well as preliminary orthotopic tumor models and the more sensitive analysis of metastatic capacities of these cells by *in vivo* bioluminescence and *in vitro* Luciferase assay, respectively. Thus, the subcutaneous model was used for intravenous bolus treatment with doxorubicin as an exemplary drug to first of all demonstrate the basic applicability of *in vivo* imaging to follow up therapeutic intervention in this PCa tumor model.

Additionally, it has been demonstrated in experimental animal models that various drugs (e.g., doxorubicin, taxanes) might display therapeutic efficacy by other mechanisms [16-22] than directly damaging tumor cells, even when PCa cells display MDR and, therefore, are primarily insensitive to the drugs themselves. Thus, another point of interest was to investigate the applicability of the models to study the basic principles of new metronomic therapies using a recently developed piezoelectric silicon micropump [23].

## 2. Results

### 2.1. Growth Characteristics and Metastasizing Capacity of Subcutaneous MAT-Lu ELN Tumors

**Figure 1.**
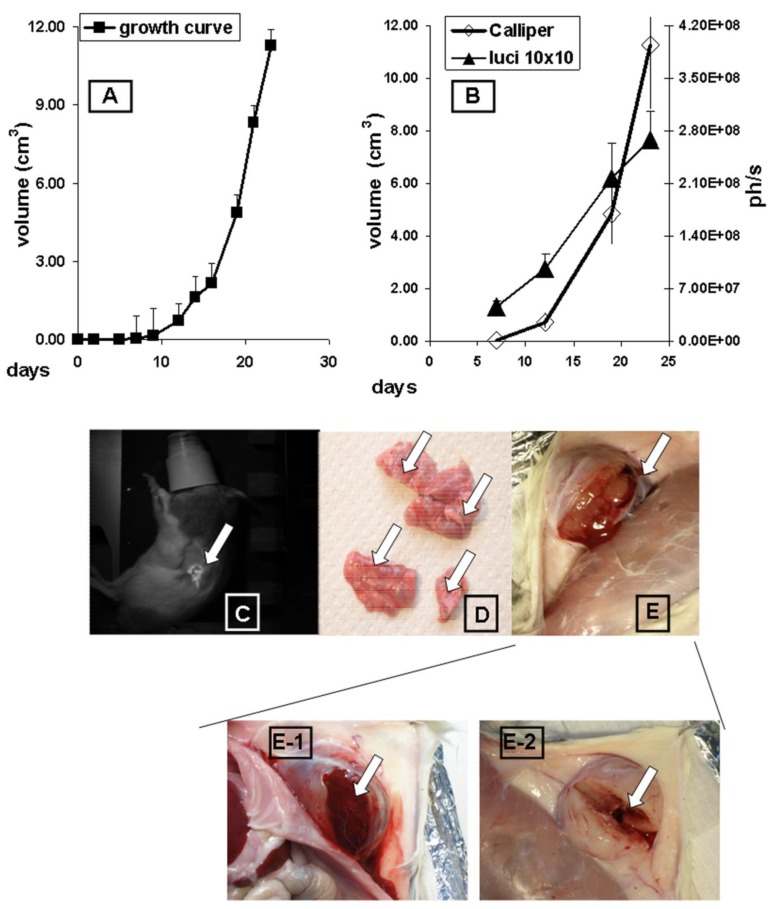
Growth characteristics of subcutaneous MAT-Lu PCa. Tumor growth was followed up every other day by callipering (**A**) and terminated at day 24. *In vivo* bioluminescence image-analysis (**B**: ▲) once a week in a Nightowl LB981 camera system (Berthold, Bad-Wildbach, Germany) shows a significant correlation to the tumor volume determined by callipering (**B**: ◊). Overlays of *in vivo* images and rat photographs (**C**). Infiltrated lungs with clearly visible metastases (**D**). Cystic structure of the subcutaneous MAT-Lu tumors (**E**), characterized by mucous necrotic liquid (E 1) and a rather loose inner structure (E-2).

The growth of subcutaneous implanted MAT-Lu ELN PCa was followed up every other day by callipering (Figure 1A). After a lag phase of about 10 days the tumors displayed fast growth which was terminated at day 24 at final tumor dimensions of 2.67 ± 0.87 × 2.08 ± 0.55 (corresponding to a volume of 11.3 ± 1.4 cm^3^ and about 5% of the weight of the rat). There was no difference in growth between MAT-Lu JHU-4 (ATCC, Johns Hopkins University Special Collection) and MAT-Lu ELN (luciferase-transduced) cells *in vivo* (data not shown), but the latter showed a slightly reduced take rate varying from 80–90% compared to non-transduced cells (100%). This was not investigated in more detail but might result from immune reactions against neo-antigens caused by the transduced protein.

Correlation between callipered tumor volume in the rats and *in vivo* bioluminescence was determined by parallel image-analysis (Figure 1B) once a week. Comparing the four time points (total growth period 24 days), Figure 1B shows a striking parallelism between *in vivo* imaging curves (ph/s) and the tumor volume determined by callipering (cm^3^) with a high correlation coefficient (R = 0.978; p < 0.0221).

The overlays of *in vivo* images and rat photographs allowed to localize primary tumor areas—here subcutaneously in the left flank—but detected no signs of metastatic lesions in other tissues of untreated animals, even in late images (Figure 1C), although the lungs were infiltrated in some cases by clearly macroscopically visible metastases (Figure 1D).

**Figure 2.**
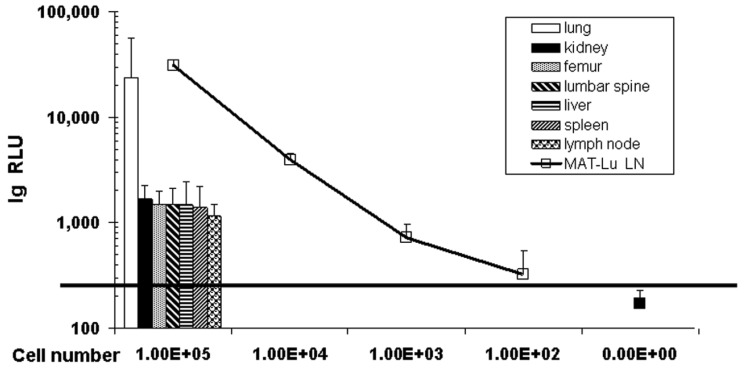
Metastatic burden in different rat tissues compared to luciferase activity in MAT-Lu ELN cells. Metastatic burden in individual tissues was determined by homogenization and subsequent luciferase assay. Metastases appear in lung, lymph nodes, spleen, kidney, liver, and bone tissue (femur or lumbar spine) of untreated animals (columns). The metastatic burden in respective tissues (columns) is shown at the y-axis as mean RLU (relative light unit) values of tissues from metastatic animals. Corresponding cell numbers were calculated by a standard curve of crude lysates from defined numbers of MAT-Lu ELN cells (□) numbered on the x-axis. The black horizontal line marks the detection limit for metastatic lesions in individual tissues which was about 100 cells per tissue piece when defined as ≥ 1.6 times of the background value (■).

The consistence of the subcutaneous MAT-Lu tumors was characterized by a rather cystic structure with a “solid tumor envelope” (Figure 1E) filled with a mucous necrotic liquid to a varying degree (Figure 1E-1), whereas the remaining inner structure was rather loose (Figure 1E-2). The greater variation of the final imaging (day 23) probably results from this varying consistence of the tumors and their different degree of necrosis.

Despite the failure to detect metastases by *in vivo* imaging, the transduction of MAT-Lu cells allowed highly sensitive tumor cell detection *in vitro* in tissue homogenates by subsequent luciferase assay [13,24], even in lung tissue with no macroscopically visible metastasis. These data not only show (Figure 2: columns) the typical metastatic infiltration of the lung by subcutaneous MAT-Lu PCa cells (13/24), but metastatic lesions could also be detected in inguinal lymph nodes (11/24), spleen (3/24), kidney (4/24), liver (5/24), and bone tissue (femur or lumbar spine - 5/20 and 12/20, respectively). In all these latter tissues, however, no metastases were ever visible. This was not surprising since metastatic burden of the latter tissues, corresponding to 2,000–6,000 metastatic cells, was only ≤1/10–1/100 of the lung (70,000–120,000 metastatic cells) (Figure 2: curve) in the individual tissues, as shown by the significantly lower RLU (relative light unit) values (Figure 2: columns).

### 2.2. Growth Characteristics and Metastasizing Capacity of Orthotopic MAT-Lu Tumors

**Figure 3.**
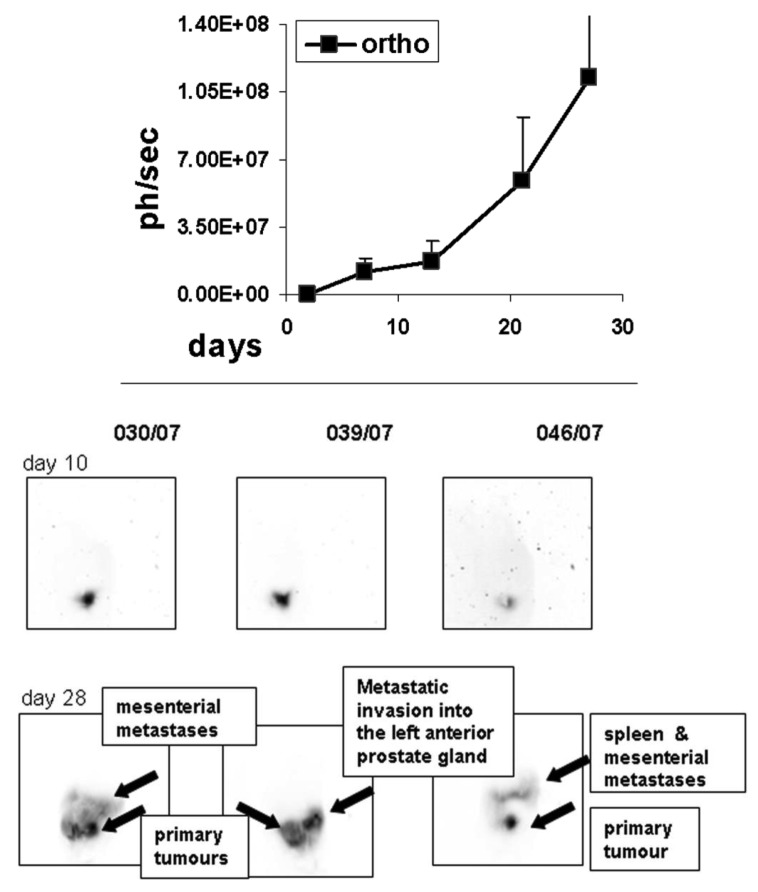
Growth curve of orthotopic MAT-Lu PCa determined by luciferase *in vivo* imaging and tumor images from orthotopic MAT-Lu PCa. (Above) Tumor growth follow up by *in vivo* bioluminescence image-analysis once a week in a Nightowl LB981 camera system (Berthold, Bad-Wildbach, Germany). Growth was terminated at day 27 due to general health situation. (Below) Inverted images of *in vivo* bioluminescence from three individual rats at different imaging time points (days 10 and 28).

To determine the growth of orthotopic rat MAT-Lu ELN PCa, we implanted the cells intraprostatic into three animals as a preliminary experiment, and followed up tumor increase by *in vivo* imaging once a week. As seen in Figure 3, tumor growth could be detected for the first time by bioluminescence seven days after implantation, while first imaging (day 2) did not show any identifiable signals. The tumor growth showed similar characteristics to the ectopic situation with weak growth during the first phase (until day 14) followed by striking exponential growth (until termination day 27). Final tumor dimensions at this time point were 4.33 ± 1.31 × 3.49 ± 0.91 cm (corresponding to a volume of 21.8 ± 5.1 cm^3^ and about 10% of the weight of the rat). The consistence of the orthotopic MAT-Lu tumors was similar to subcutaneous ones (see above) and also of rather cystic structure (not shown). No body weight reduction was observed (data not shown).

Inverted orthotopic images of *in vivo* bioluminescence from individual rats at the different imaging time points show dramatic changes and further invasion into other tissues (not observed in ectopic tumors), e.g., the opposite arm of prostate gland, into the spleen, or the mesentery which could be visualized in the late phase of tumor growth beginning with day 25 (not shown) and then at day 28 (Figure 3, Figure 1 supplementary information).

In contrast to tumor growth and consistence, metastasizing capacity of orthotopic MAT-Lu tumors differed remarkably from that of subcutaneously implanted tumor cells (Figure 2). In the case of orthotopic implantation, the metastatic spread (Table 1) shows a pattern with major invasion into lymph nodes, spleen or stomach, whereas the lung was infiltrated to a lesser degree (corresponding to only about 500–2,000 cells per lung). Metastatic burden in bone tissue was similar to the ectopic situation but observed in all three animals, both in femur and lumbar spine, compared to only 25% or 60% of animals in the subcutaneous setting. Because of the small number of orthotopically implanted animals, however, these results will have to be confirmed in further studies.

### 2.3. Determination of the IC_50_-values of Doxorubicin in Non-Transduced MAT-Lu JHU-4 and Transduced MAT-Lu ELN in vitro

First, we compared transduced MAT-Lu ELN and parental MAT-Lu JHU-4 cells and determined their sensitivity to various chemotherapeutic drugs (doxorubicin, hexadecylphosphocholine, and gemcitabine) *in vitro*.  Figure 2 (supplementary information) shows a direct comparison of the two MAT-Lu cell lines treated with varying concentrations of doxorubcin. There was no significant difference between the transduced and non-transduced cells. The IC_50_-values of doxorubicin were 82.9 ± 6.5 nM for transduced and 74.5 ± 5.5 nM for non-transduced cells.

### 2.4. Anti-Tumor and Anti-Metastatic Effects of Intravenous Bolus Doxorubicin Application in the Subcutaneous MAT-Lu Model

After establishing the rat models we first assessed the use of the subcutaneous MAT-Lu PCa model for chemotherapeutic intervention. We decided to use doxorubicin as an exemplary drug to follow up the efficacy of treatment by *in vivo* imaging, particularly with respect to a future metronomic therapy using a recently developed piezoelectric silicon micropump (see below).

There were several reasons for this decision. First of all, doxorubicin was found to be very stable—up to 4 weeks at 37 °C—in the pump reservoirs as determined by HPLC-analysis (Figure 3 supplementary information). Furthermore, doxorubicin was already successfully applied in various metronomic *in vivo* studies [19,32,33].

**Figure 4.**
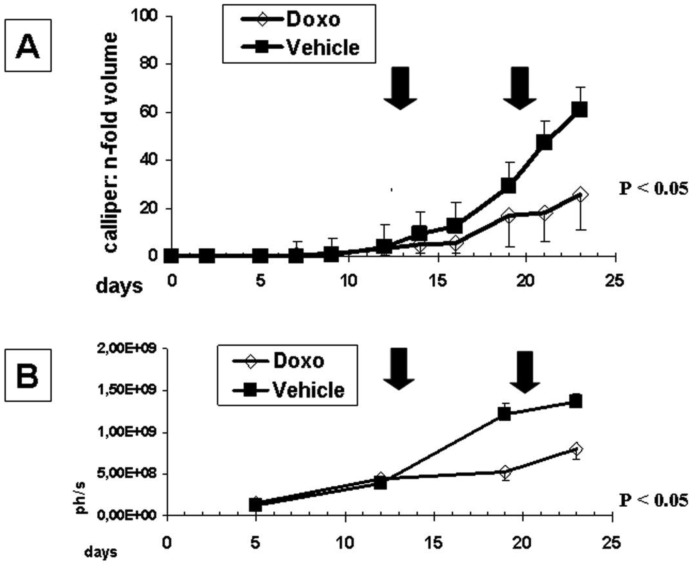
Anti-tumor effects of intravenous bolus doxorubicin application in the subcutaneous MAT-Lu model (1×10^6^ cells). Starting with day 13 (palpable tumors: 0.80 ±0.37 × 0.57 ± 0.21) the rats were treated (arrows) two times once weekly with 1 mg/kg doxorubicin or the vehicle (NaCl). At day 23 a significantly (p = 0.0131) reduced subcutaneous tumor growth could be observed compared to the vehicle control (10.3 ± 2.6 cm^3^). Data refer (**A**) to n-fold tumor volume determined by callipering or (**B**) to bioluminescence (ph/sec) in seven animals (one animal died for other than experimental reasons) in the doxorubicin and eight animals in the control group.

Using bolus of two times 1mg/kg doxorubicin once a week we could significantly (p = 0.0131) reduce subcutaneous tumor growth compared to the vehicle control either determined by callipering (Figure 4A) or *in vivo* imaging (Figure 4B).

Doxorubicin not only inhibited primary tumor growth but also reduced the number of metastatic animals as well as the metastatic burden in individual tissues (Table 2). However, whereas the animal group size was large enough to analyze anti-tumor effects of doxorubicin treatment, a direct statistical comparison of results regarding metastatic burden in the various tissue types was—with the exception of lymph nodes—impossible, because of the small number of affected animals (ranging from 12%–75%).

However, comparing either the total number of animals affected by metastatic lesions (16/48 untreated *versus* 7/41 treated animals) or the mean of metastatic burden in all tissues (*i.e.*, mean RLU of all tissues: 101,016 ± 30,410 *versus* 52,198 ± 18,087) from animals without and with doxorubicin treatment, respectively, significantly reduced metastatic lesions (p = 0.0291) as well as metastatic burden (p = 0.0394) were found in the paired t-test.

### 2.5. MAT-Lu ELN Tumors as Model for Metronomic Intravenous Doxorubicin Application Using a Piezoelectric Silicon Micropump

In the next step, the subcutaneous model was used in slightly modified form (5 × 10^5^ instead of 1 × 10^6^ cells were implanted to prolong growth period from 23 to 32 days) for preliminary investigation of basic principles of metronomic doxorubicin therapy via jugular vein catheter using a newly established active microport pumping system. As already mentioned above, a high stability of doxorubicin in the storage chamber of the pumping system has been demonstrated (Figure 3 supplementary information).

To in principle establish the technical applicability of the active microport pumping system for metronomic therapy in this model, various experimental problems had to be solved previously, *i.e.*, the successful catheterization via jugular vein, the permanent patency of the heparinized port, and the continuous infusion of the drug.

We found that catheter implantation—as described in Experimental Section—did not cause any problems in the animals concerning wound healing or infections of surgical lesions. The fixed jugular vein catheter was rinsed after each use (since it was not used continuously) with about 100 μL of 50 IU heparin per mL in physiological saline and thereby remained permeable for at least 22 days after implantation in the nine animals investigated.

**Figure 5.**
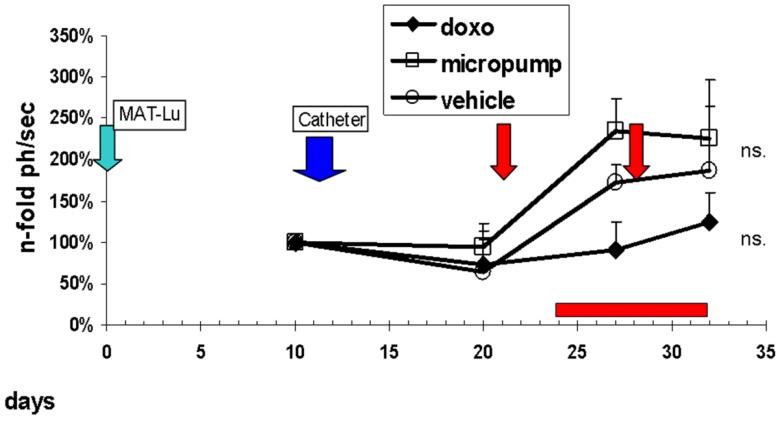
Anti-tumor effects of intravenous metronomic micropump-mediated doxorubicin application in the subcutaneous MAT-Lu model. Tumor growth was followed up by *in vivo* bioluminescence image-analysis in a Nightowl LB981 camera system (Berthold, Bad-Wildbach, Germany) starting with day 10 after tumor implantation (green arrow). Catheter embedment was performed between days 12 and 14 (blue arrow) in 9 animals. Metronomic application (micropump) of daily 0.1 mg/kg doxorubicin (□) was given from day 24 until day 32 (red bar at bottom). The experiment was terminated on day 32 after tumor implantation (final tumor volume 17.3 ± 4.6 cm^3^). At day 21, followed by day 28, control treatments (red arrows) were started with bolus injection of 1mg/kg doxorubicin (♦) in 9 animals or the vehicle (○) in 10 animals (these groups were not sham operated). Green arrow (MAT-Lu) = tumor implantation; blue arrow (Catheter) = catheter embedment; red arrows = doxorubicin bolus injection; red bar = metronomic doxorubicin application by micropump

Metronomic application of doxorubicin to rats in our studies was performed either via an external computer-guided pump using a universal Swivel-to-Tether infusion system (Harvard Apparatus, Holliston, MA) (Figure 4 supplementary information Part 1) or an autonomous pumping system with integrated control module to jugular vein catheters (Figure 4 supplementary information Part 2). The autonomous pumping system was directly fixed to the animals near the catheter port using rat jackets (Figure 4 supplementary information Part 2). The preliminary dose of 0.1 mg/kg [25] was given daily for seven days. Infusion time per animal via external or autonomous pumping system, however, varied only between 30–60 min each (Figure 5).

As seen from the tumor growth curves (Figure 5), preliminary daily metronomic dose of 0.1 mg/kg (□) did not affect tumor growth and resulted in a very similar course as observed in the vehicle control group (○). Intravenous doxorubicin bolus application (♦) caused a pronounced inhibition of tumor growth, but in contrast to the earlier study (Figure 4), this effect was also not significant, probably resulting from the later (days 21/28 *versus* days 15/22) therapeutic doxorubicin application, caused by technical reasons of the catheter implantation in the metronomic application group.

## 3. Discussion

One goal of our present study was to establish well-characterized, clinically relevant models, *i.e.*, metastasizing, hormone-refractory rat prostate cancer (HRPCa) cells suitable for ectopic as well as orthotopic implantation. Recently, the stable genetic labeling of tumor cells with markers such as GFP or luciferase has offered the possibility of simple and non-invasive follow up not only of palpable ectopic (e.g., subcutaneous) tumors but also of visceral orthotopic tumors *in vivo* (e.g., in prostate or pancreas) [13,15,26,27]. Additionally, it significantly ameliorated *in vivo* or *in vitro* localization of metastases [14,28-30]. We have shown here that the luciferase-transduced MAT-Lu animal models can be applied for the development and investigation of new systemic therapies [1] and to study factors that regulate metastatic outgrowth. The MAT-Lu ELN cells established here allowed to follow up orthotopic PCa growth, to compare metastasizing capacity of ectopic as well as orthotopic tumors, and to quantify metastatic burden in individual tissues with and without therapy.

Chemotherapy is the standard treatment option for advanced disease [2-4]. However, HRPCa often become resistant to various chemotherapeutic drugs and this multidrug-resistant nature minimizes the effectiveness of such therapies. New treatment strategies, e.g., the application of liposomally entrapped drugs [13], the use of less toxic pro-drugs [31], or metronomic, *i.e.*, continuous low-dose drug application [19,25,32], seem to enhance the effectiveness of treatments even with drugs which do not or not anymore show direct efficacy in multidrug-resistant tumors in experimental cancer and clinical treatment [33,34].

MAT-Lu tumors are a metastasizing variant of the Dunning R3327 Copenhagen rat prostatic carcinoma which, when implanted subcutaneously in the flank or intramuscularly in the hind leg, invariably metastasise into the lungs [11]. Nevertheless, here, using the highly sensitive tumor cell detection by *in vitro* luciferase assay, we could show metastatic lesions in other tissues as well (lymph nodes, spleen, kidney, stomach, or bone tissue). The metastatic invasion of these tissues compared to lung, however, occurred to a remarkably lesser degree (only about 1/10–1/100), concerning the metastatic burden per tissue but also the number of animals with metastatic lesions.

In contrast, orthotopic tumor growth displayed a clearly different pattern of metastatic spread with preferential invasion into inguinal lymph nodes, spleen, and stomach. Due to the small number of animals tested until now in the orthotopic approach, however, we cannot completely exclude any leakage of cancer cells into the peritoneal cavity, which could cause peritoneal carcinosis (a potential “leakage tumor growth”) and consequently mislead to conclude that there actually were metastases in the mesentery, spleen or stomach. But, with respect to lymph node and bone invasion the differences in metastasizing capacity of the two application forms are notable. Orthotopic implantation resulted in striking invasion into lymph nodes and metastases were found in femur and spine cord tissue in all cases (Table 1 and Figure 2), rather comparable to the human metastatic pattern [35]. However, as mentioned above, these data also have to be confirmed in a larger number of animals.

Another difference between ectopic and orthotopic implantation had consequences for *in vivo* imaging (Figure 3, Figure 1 supplementary information). The probably higher tumor cell burden in endometrium or spleen could be detected and followed up by respective bioluminescence signals in later phases of orthotopic tumor growth (Figure 3, Figure 1 supplementary information). On the other hand, the macroscopically visible lung metastases were not detected in the ectopic situation by *in vivo* bioluminescence (Figure 1). This difference to subcutaneous tumors might exist for various reasons. Most importantly, the brightness of the signal in orthotopic tumors, although in the end (about two times) stronger than in subcutaneous ones (see Figure 1B and Figure 3), was only about one third to a half of the signals compared to the subcutaneous tumor, probably due to “quenching” of the signals by other visceral tissues (see: Figure 1 supplementary information B). Thus the detection of weaker signals also became possible (Figure 3).

The other goal of the study was to examine basic principles of new therapies using a recently developed piezoelectric silicon micropump [23]. We could successfully demonstrate the technical applicability of the models for metronomic therapy, *i.e.*, the catheterization via jugular vein, the permanent patency of the heparinized port, and the continuous infusion of the drug via an external computer-guided pump using a universal Swivel-to-Tether infusion system (Harvard Apparatus, Holliston, MA) or an autonomous pumping system with integrated control module, over a time period of 20 days (see also supplementary online material).

Although the bolus control treatment with doxorubicin did already inhibit primary tumor growth as well as metastatic spread after two intravenous bolus treatments (Figure 4, Table 1), a failure of micropump-mediated metronomic intravenous doxorubicin therapy in the subcutaneous model (Figure 5) was observed. This might result from various causes: (i) using only a “reference concentration from literature” [25]; (ii) a discontinuous (within 30–60 min); and (iii) late-onset drug application (beginning with days 21 for bolus, or 24 for metronomic treatment); as well as iv) an overall longer tumor growth period (32 days) with about 1.6 times larger final tumor volumes compared to the first bolus application. The latter two points also might be responsible for the reduced, insignificant effects of bolus doxorubicin in this application.

However, therapeutic efficacy was not the primary question of the presented experiments. We used this approach to verify the principle technical suitability of the pumping system [23], and the luminescent MAT-Lu ectopic and orthotopic tumors to be valuable models, rendering the possibility for investigation of new therapeutic applications.

## 4. Experimental Section

### 4.1. Cell Culture and Cell Lines

Hormone-refractory rat prostate carcinoma (PCa) cell lines MAT-Lu (ATCC, Johns Hopkins Special Collection: JHU-4) cells were routinely passaged *in vitro* in DMEM supplemented with 1% Glutamine, 1% Pen-Strep, 1% Fungizone (Invitrogen, Heidelberg, Germany), 250 nM Dexamethasone (Fortecortin 4 mg, Merck KGaA, Darmstadt, Germany) and 10% FCS (Cambrex, Verviers, Belgium) at 37 °C with 10% CO_2_ in a humidified atmosphere.

### 4.2. Generation of Luciferase Expressing MAT-Lu PCa cells

The retrovirus encoding the Luciferase-aminoglycoside phosphotransferase (Neomycin resistance) fusion gene (Luci-Neo) was constructed from the luciferase gene of pUHC 13-3 (pTRE Luc, [36]), and the neomycin resistance gene from pcDNA 3.1 (Invitrogen, Heidelberg, Germany), using pLib (BD Clontech, Heidelberg, Germany) as a backbone. The EF1α was derived from a pEF vector (Invitrogen, Heidelberg, Germany), and introduced upstream of the Luci-Neo fusion gene. The transduction of the MAT-Lu cells using a VSV-G (BD Clontech, Heidelberg, Germany) pseudotyped retrovirus was performed according to the instructions from the manufacturer. After selection of successfully transduced MAT-Lu ELN cells using 1mg/mL Neomycin, their luciferase activity was tested. 10^6^ cells were lysed in 100 μL in 1 × luciferase lysis buffer (25 mM TRIS-phosphate pH 7.8; 2 mM EDTA; 2 mM DTT; 0.1% Triton X-100), the lysate was serially diluted, and assayed for luciferase activity (Promega E4550), according to the manufacturer's instructions in a Luminometer (BMG Lumistar).

### 4.3. Determining the IC_50_ of Doxorubicin in Non-Transduced MAT-Lu JHU-4 and Transduced MAT-Lu ELN in vitro

100 μL of the cells were seeded at 1 × 10^5^/mL per well into 96 well plates (Greiner BioOne, Frickenhausen, Germany). After 24 h, another 100 μL of doxorubicin were added at indicated final concentrations and the cells were incubated for another 48 h. Finally, BrdU reagent (Roche Diagnostics GmbH, Penzberg, Germany) was added for 4 h. Culture supernatants were removed, the cells were fix-dried for 1 h at 60 °C, and stored at 4 °C. BrdU assays were performed according to the manufacturer's instructions.

### 4.4. Animal Experiments

All animal experiments were performed in accordance with German Animal License Regulations (Tierschutzgesetz) identical to UKCCCR Guidelines for the welfare of animals in experimental neoplasia [37]. Copenhagen (COP/NCrl) rats were obtained from Charles River Laboratories (Sulzfeld, Germany). Schematic representation of animal experimental protocol is shown in Table 1 supplementary information.

Subcutaneous MAT-Lu ELN PCa tumors were induced by injecting 1 × 10^6^ or 5 × 10^5^ cells in 50 μL DMEM (Invitrogen, Heidelberg, Germany) per animal subcutaneously into the left flank of COP rats. Tumor sizes were measured three times weekly via callipering and compared once weekly by Luciferase imaging. Tumor volume was calculated by the formula A × B × A/2, where A is the major and B is the minor diameter. The rats were killed when the tumor weight had reached 10% of the body weight (as calculated from tumor volume) of the rat or when the tumors started to ulcerate.

Orthotopic MAT-Lu ELN PCa tumors were induced by injecting 1 × 10^6^ cells per animal in 25 μL DMEM (Invitrogen, Heidelberg, Germany) into the left or right anterior prostate gland of COP rats. Successful implantation and tumor growth was monitored by *in vivo* bioluminescence (see below). Stop criteria in the orthotopic model were the general health situation (scrubby coat) and/or body weight loss (>20%) of the animals.

### 4.5. Active Microport and Jugular Vein Catheterization

A 1 to 1.5 cm skin incision was made over the ventral thorax slightly left or right of the centre. Another 1 to 1.5 cm skin incision was made in the neck of the animals. A portion of the vein was freed from all underlying tissue to visualize the jugular vein as it passes under the left or right clavicle into the chest cavity. After isolating an approximately 5 mm section of the vein; two fine silk ligatures were placed at either end of the isolated section of the vein. The ligature closest to the head was tied to occlude blood flow going to the heart. The venotomy was carefully performed in the isolated section of the vein close to the tied cranial ligature with micro scissors. The Funnel-Cath mouse catheter (BS4 72-4441, Harvard Apparatus GmbH, Germany) was filled with 50 IU Heparin per mL (Sigma, H0880) in physiological saline and closed at the catheter port by a small steel plug. The tip of the catheter was then advanced into the lumen of the vein. After crossing the lower ligature the catheter was advanced for a further 5 mm and fixed closing both ligatures. Additionally, a third silk ligature was placed between the two others to stabilize the fixation. A small trocar was used to form a subcutaneous tunnel from the neck to the thoracic incision. The catheter port was then advanced through the trocar lumen to the neck incision. Here a Dacron mesh button tether (BS4 61-0034. Harvard Apparatus GmbH, Germany) was implanted to establish a long-term catheter in- and outlet and the catheter port was secured with a clip at the button tether. Functionality of the catheter was tested after opening the catheter port by carefully aspirating and expelling blood with heparin physiological saline syringe. The latter will ensure that all blood aspirated into the catheter tip during implantation is expelled.

### 4.6. Measurement of in vivo Bioluminescence

300 μL of the substrate, D-Luciferin (20mg/mL; Synchem OHG, Germany), were injected i.p. in two portions of 150 μL, respectively, and the animals were then anesthetised in an isofluorene chamber. Ten minutes later they were transferred into the Nightowl LB981 (Berthold, Bad-Wildbach, Germany), and exposed for 1 min at 2 × 2 binning, and 5 min at 10 × 10 binning. For quantification of light signals bioluminescence was analyzed on raw images from the camera using the internal software “WinLight32” (Berthold, Bad-Wildbach, Germany) and expressed as photons/second.

### 4.7. Quantification of Metastatic Lesions by in vitro Luciferase Assay

To screen and quantify metastatic lesions (endpoint analysis) in potential target organs of rats, pieces of lung, liver, spleen, inguinal lymph node, stomach, kidney, femur, or lumbar spine were homogenized in 1 mL (liver in 5mL) of luciferase lysis buffer (CCLR) using a tissue homogenizer Fastprep-24 and Lysing MatrixA tubes (MP Biomedicals, Heidelberg, Germany). Insoluble material was spun down 10 min at 15,000 rpm in a Heraeus Biofuge15. 5 μL of the supernatant were checked for protein concentration using a Bradford assay (Sigma B6916) with BSA serving as a standard protein, and 10 μL were measured in a luciferase assay (Promega E1501). Data are shown as log RLU (relative light units) normalized by protein concentration.

### 4.8. Statistics

Statistical analyses were performed using student's t-test or—if Normality Test failed—using Mann-Whitney Rank Sum Test (SigmaStat 3.1).

## 5. Conclusions

In summary, our data clearly show that luciferase expressing rat MAT-Lu cells are useful as ectopic, as well as orthotopic autologous PCa, models in immune competent animals, and allow to sensitively analyze the metastatic behavior of these cells. With regard to the piezoelectric silicon micropump [23] with a pump capacity of 0.1–50 μL per minute, useable as an implantable active microport [38], we could show its suitability for the metronomic application of drugs.

Thus the described models allow the investigation of new anti-tumor and anti-metastatic therapeutic approaches, such as micropump-mediated metronomic chemotherapy, the use of prodrugs [31] or the application of liposomally entrapped drugs [13].

## Figures and Tables

**Table 1.** Metastatic burden in different rat tissues of orthotopic MAT-Lu PCa determined by *in vitro* luciferase assay.

	**LN**	**SP**	**KI**	**ST**	**LI**	**LU**	**FE**	**LS**
**ortho**	**46,441** ± 3,429	**14,112** ± 2,035	**4,912** ± 879	**27,495** ± 4,102	**5,807** ± 5,410	**1,277** ± 679	**1,122** ± 512	**2,470** ± 1,822

MAT-Lu cells were orthotopically implanted into three animals. The three animals developed metastatic lesions within 28 days in all investigated tissues. Means and standard deviation are shown in RLU (relative light units) normalized by protein values for LN = lymph node, SP = spleen, KI = kidney, ST = stomach, LI = liver, LU = lung, FE = femur, LS = lumbar spine. Background values without metastasis were 206 ± 56 RLU. Grey scale of the table columns corresponds to: black = ≫ 100,000 cells, grey = 50,000–100,000 cells, light grey = 500–10,000 cells per infiltrated tissue.

**Table 2.** Anti-metastatic effects of intravenous bolus doxorubicin application in the subcutaneous MAT-Lu model.

		**LN**	**SP**	**KI**	**LI**	**LU**	**FE**	**LS**
doxo*	affected animals	0/7 ^p < 0.05^	1/7	0/7	2/7	3/7	1/3	0/3
	RLU		789		708 ± 389	16,149 ± 19,775	1,546	
vehicle	Affected animals	4/8	2/8	1/8	2/8	6/8	1/4	2/4
	RLU	1,065 ± 518	1,491 ± 1,105	2,158	1,880 ± 1,701	13,895 ± 25,051	2,350	1,068 ± 754

LN = lymph node, SP = spleen, KI = kidney, ST = stomach, LI = liver, LU = lung, FE = femur, LS = lumbar spine,

* one animal died for other than experimental reasons. RLU (relative light units) = means from all metastatic animals are shown in luciferase light units (LU) normalized by protein values. A reduction of metastatic lesions by doxorubicin was found either with respect to the number of affected animals (LN, LU, LS) or to the amount -as reduced RLU- of metastatic burden (SP, LI, FE), but was found -because of the small number of affected animals- to be significant (p < 0.05) only in LN.

## Supplementary Files

Supplementary File 1 PDF-Document (PDF, 491 KB)
