# Supplementary material for: Metastasizing, Luciferase Transduced MAT-Lu Rat Prostate Cancer Models: Follow up of Bolus and Metronomic Therapy with Doxorubicin as Model Drug
Source: Cancers (Basel). 2011 Jun 17;3(2):2679–95. doi: 10.3390/cancers3022679 (PMC3757437; doi:10.3390/cancers3022679)
Supplement: Supplementary File 1 — PDF-Document (PDF, 491 KB) [file cancers-03-02679-s001.pdf]

Cancers **2011**, *3*, 2679-2695; doi:10.3390/cancers3022679

OPEN ACCESS

cancers

ISSN 2072-6694

www.mdpi.com/journal/cancers

Supplementary File

Metastasising, Luciferase-Transduced MAT-Lu Rat Prostate Cancer Models: Follow up of Bolus and Metronomic Therapy with Doxorubicin as Model Drug

Peter Jantscheff [1,*], Norbert Esser [2], Andreas Geipel [3,†], Peter Woias [3], Vittorio Ziroli [1], Frank Goldschmidtboing [3] and Ulrich Massing [1]

[1] Tumour Biology Center, Clinical Research, Department Lipids & Liposomes, Breisacher Str.117, D-79106 Freiburg, Germany; E-Mails: ziroli@tumorbio.uni-freiburg.de (V.Z.); massing@tumorbio.uni-freiburg.de (U.M.)

[2] ProQinase GmbH, Breisacher Str.117, D-79106 Freiburg, Germany; E-Mail: n.esser@proqinase.com

[3] Laboratory for Design of Microsystems, Department of Microsystems Engineering (IMTEK), Georges-Köhler-Allee 106, D-79110 Freiburg, Germany; E-Mails: ageipel@imtek.de (A.G.); woias@imtek.de (P.W.); fgoldsch@imtek.de (F.G.)

† Present address: Roche Diabetes Care AG, Kirchbergstrasse 190. CH-3401 Burgdorf, Switzerland

* Author to whom correspondence should be addressed; E-Mail: jantscheff@tumorbio.uni-freiburg.de; Tel.: +49-761-206-1880; Fax: +49-761-206-261-1880.

Received: 4 May 2011; in revised form: 16 May 2011 / Accepted: 16 June 2011 / Published: 17 June 2011

Figure 1supp. Tumour localisation in orthotopic MAT-Lu PCa. Overlays of *in vivo* images and rat photographs allow localisation of primary tumour areas in the left anterior prostate gland of COP rats (A: blue arrow). After orthotopic implantation, metastatic lesions (see also Figure 3) in other tissues (here spleen and the mesentery) of the animals could also be visualised (A: white arrow) by in vivo imaging. Tumour localisation was confirmed by necropsy (B: blue arrow) and metastases in the mesentery were also macroscopically visible (not shown). The primary PCa tumour (B: blue arrow) is covered by other visceral tissues.

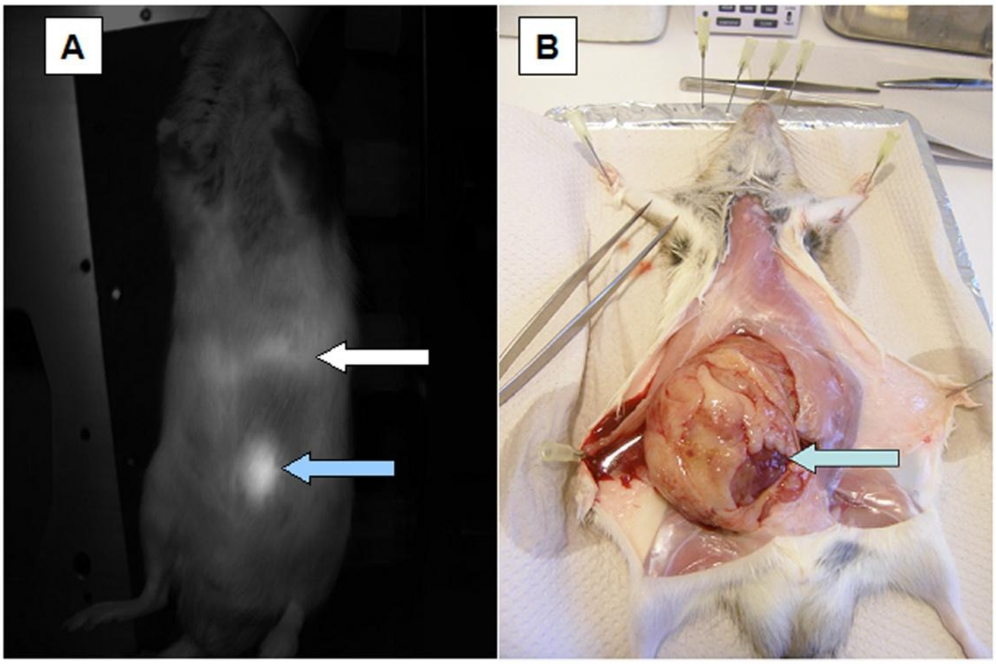

Table 1supp. Schematic representation of animal experimental protocols.

	Vehicle Group	Bolus Group	Pump-Group
**Growth curve** (ectopic)	6[&]		
**Bolus treatment***	8	7	
**Metronomic treatment****	10	9	9
**Growth curve** (orthotopic)	3		

Growth curves were determined in 6 and 3 animals, respectively, for ectopic or orthotopic application without any treatment. * Bolus treatment was performed with 1mg/kg doxorubicin on days 13 and 20 after subcutaneous tumour inoculation (1×10^6 cells) in 8 (vehicle) or 7 (doxorubicin) animals. ** Metronomic treatment was performed with 0.1 mg/kg applied - for technical reasons - within 30–60 minutes in 9 animals. As a control, 9 animals were treated with bolus injection of 1 mg/kg doxorubicin and 10 animals with vehicle on days 21 and 28 after subcutaneous tumour inoculation (5×10^5 cells). [&]animal number of individual treatment groups.

Figure 2supp. IC_{50} values of doxorubicin in non-transduced MAT-Lu JHU or transduced ELN PCa cells in vitro. MAT-Lu JHU and transduced MAT-Lu ELN PCa cells were incubated with varying concentrations of various drugs for 24 to 120 h. Optimal logarithmic growth of the cells was seen at about 48 h. Results from 3 independent experiments are shown as percentage of proliferation (BrdU incorporation) of the vehicle control (NaCl) at 48 h. For doxorubicin, the IC_{50} values were very similar in transduced 82.9 ± 6.5 as well as, despite of transduction and selection with G418 (1 mg/mL), non-transduced cells 74.5 ± 5.5 nM. The IC_{50} of gemcitabine in MAT-Lu ELN cells (data not shown) was 9.6 ± 2.3 nM, which might be an indirect reference to the MDR+ phenotype of MAT-Lu cells, since gemcitabine (20, 20-difluoro-20-deoxycytidine, dFdC) recently was shown to display increased efficacy to cell lines from several tumor entities, which display a doxorubicin-resistant MDR+ phenotype [1].

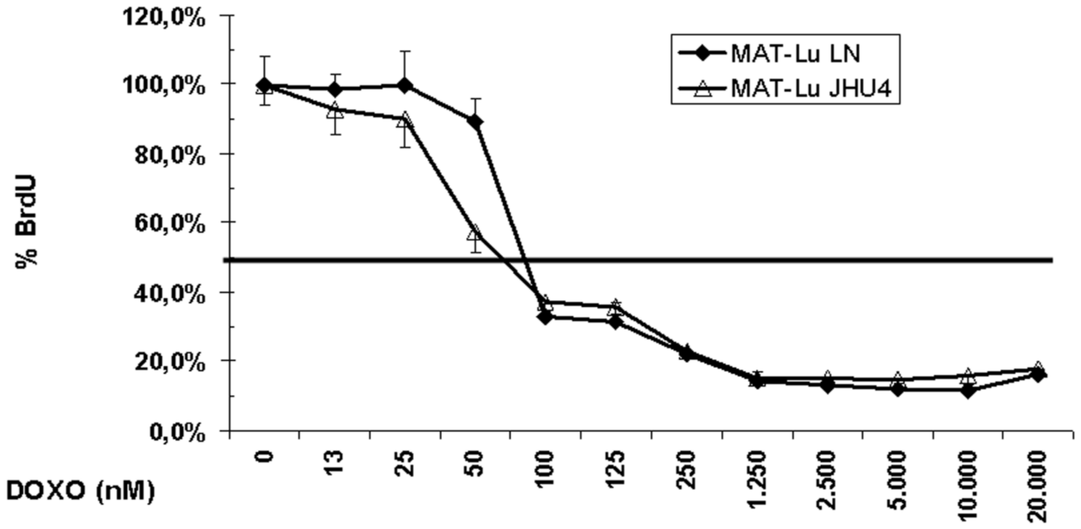

Supplementary Results & Discussion

A model for metronomic intravenous doxorubicin application using a piezoelectric silicon Micropump

Recently, we have developed a piezoelectric silicon micropump [2] with a pump capacity of 0.1–50 µL per minute useable as an implantable active microport for metronomic application of drugs [3].

One point of interest with respect to long-term applicability of the pumping system for a metronomic therapy was the stability of respective drugs in the drug reservoir of the micropump. We investigated stability of doxorubicin by HPLC degradation analysis (Figure 3supp A, B). For this purpose, the drug was stored in the pump reservoir at body temperature (37 °C), room temperature, as well as at 4 °C for various lengths of time (Figure 4supp C). The obtained data show that doxorubicin is sufficiently stable at 37 °C for a long-term application. Other drugs (e.g., gemcitabine) were not as stable at working solution (data not shown). Thus, doxorubicin was selected for a preliminary metronomic implementation of the microport system.

To use the new active microport pumping system, first of all various technical problems had to be solved to demonstrate in principle the technical applicability of the pump for metronomic therapy in this model, *i.e.*, the successful catheterisation via jugular vein, the permanent patency of the

heparinised port, and the continuous infusion of the drug via an external computer-guided pump. As shown in figure 4supp the latter was tested either using a universal Swivel-to-Tether infusion system (Harvard Apparatus, Holliston, MA, USA) or an autonomous pumping system with integrated control module. We found that catheter implantation - as described in the Experimental Section - did not cause any problems in the animals concerning wound healing or infections of surgical lesions. The fixed jugular vein catheter was rinsed after each use (since it was not used continuously) with about 100 µL of 50 IU heparin per ml in physiological saline and thereby remained permeable for at least 22 days after implantation in the nine animals tested.

Figure 3supp. HPLC analysis of drug stability in the micropump reservoir. Drug stability was determined for 150 days and a valuable stability (≥ 90%) was seen at body temperature (37 °C) up to day 70, whereas at room temperature or at 4 °C doxorubicin was stable ≥150 days. A and B show HPLC diagrams of 0.2 mg/ml doxorubicin stored 8 weeks at 4 °C and 37 °C, respectively. The left peaks represent doxorubicin (RT = ~8.5), whereas the right peak depicts a degradation product (RT = ~10.8). C summarizes the data of three to four independent experiments.

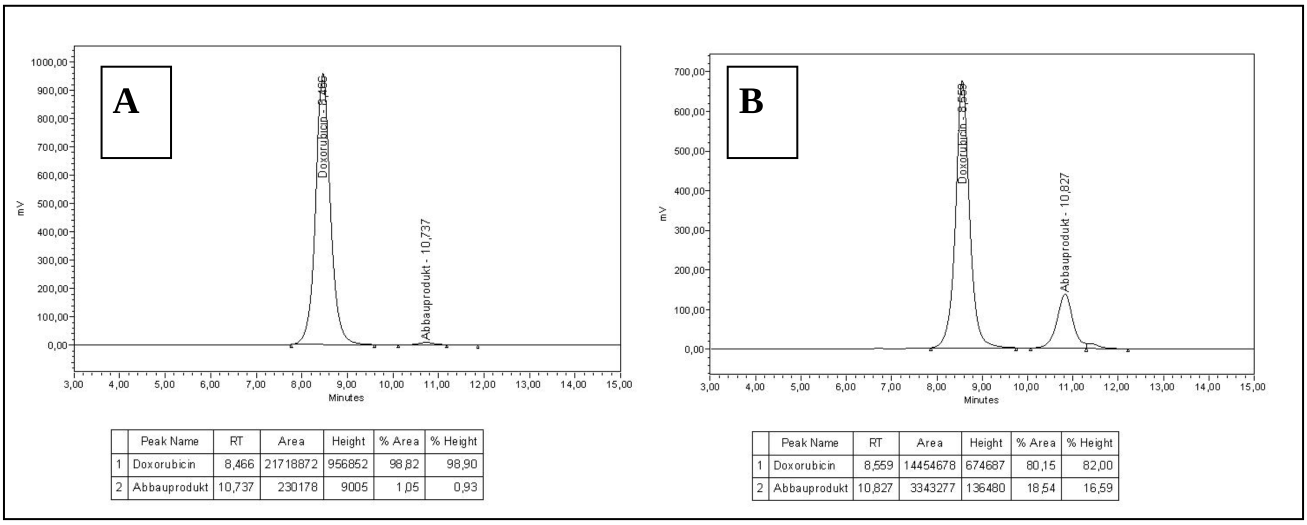

	Peak Name	RT	Area	Height	% Area	% Height
1	Doxorubicin	8,466	21718872	956852	98,82	98,90
2	Abbauprodukt	10,737	230178	9005	1,05	0,93

	Peak Name	RT	Area	Height	% Area	% Height
1	Doxorubicin	8,559	14454678	674687	80,15	82,00
2	Abbauprodukt	10,827	3343277	136480	18,54	16,59

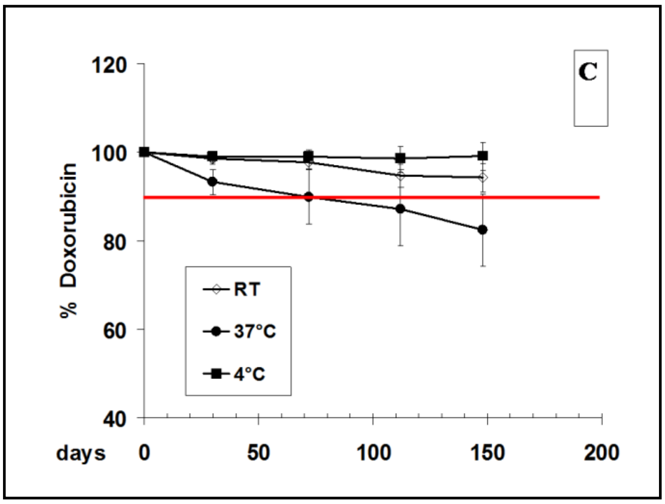

Figure 4supp. Technical solutions for metronomic doxorubicin therapy via a jugular vein catheter using a piezoelectric silicon micropump system. The new active microport pumping system for metronomic therapy was connected to the rats either as an (1) external computer-guided pump via a universal Swivel-to-Tether infusion system (Harvard Apparatus, Holliston, MA), or as an (2) autonomous pumping system with integrated control module to a jugular vein catheter. The autonomous pump system was directly fixed to the animals near the jugular catheter port using rat jackets. A = external pump, B = Swivel-to-Tether infusion system (Harvard Apparatus, Holliston, MA), C = computer-controlled guidance, D = catheter port, E = battery and integrated control module, F = rat jacket (Harvard Apparatus, Holliston, MA), and G = catheter port and active microport pumping system.

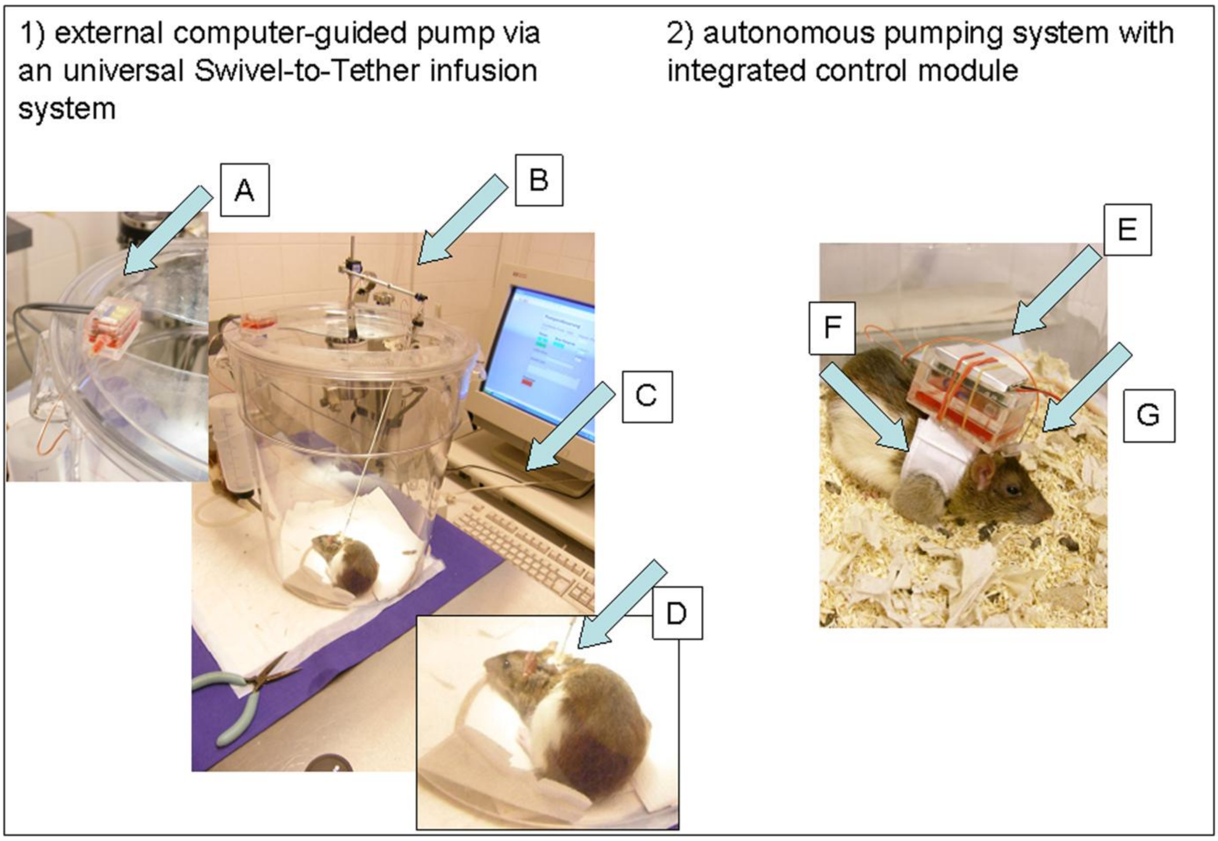

Supplementary Experimental Section

HPLC determination of doxorubicin degradation

Diluted samples (1:2) and calibrators (range 10–1000 ng/mL) were cleaned up by solid phase extraction (Oasis™ HLB 1 CC, Waters). Eluate was concentrated to 200 µL by a speedvac concentrator and Doxorubicine was quantified on a waters HPLC system (column: LiChrosphere Select B, 5 µm, 250 × 4 mm with precolumn (4 × 4mm); eluent: trietyhlamine/trichloric acid buffer (pH 2,0) / acetonitrile / methanol 66.3/22.5/11.2 (v/v/v); Flow gradient (ml/min): 1.1 (0 min.) – 2.2 (12.5 min.); Injection vol. 75 µL; fluorescence detection (ex. 481 nm, em. 550 nm)).

Supplementary References

1. Bergman, A.M.; Pinedo, H.M.; Talianidis, I.; Veerman, G.; Loves, W.J.; van der Wilt, C.L.; Peters, G.J. Increased sensitivity to gemcitabine of P-glycoprotein and multidrug resistance-associated protein-overexpressing human cancer cell lines. *Br. J. Cancer* **2003**, *88*, 1963-1970.
2. Geipel, A.; Doll, A.; Jantscheff, P.; Esser, N.; Massing, U.; Woias, P.; Goldschmidtboeing, F. A novel two-stage backpressureindependent micropump: Modeling and characterization. *J. Micromech. Microeng.* **2007**, *17*, 949-959.
3. Geipel, A.; Goldschmidtboeing, F.; Jantscheff, P.; Esser, N.; Massing, U.; Woias, P. Design of an implantable active microport system for patient specific drug release. *Biomed. Microdevices* **2008**, *10*, 469-478.

© 2011 by the authors; licensee MDPI, Basel, Switzerland. This article is an open access article distributed under the terms and conditions of the Creative Commons Attribution license (http://creativecommons.org/licenses/by/3.0/).
